# Discovery of a Good Responder Subtype of Esophageal Squamous Cell Carcinoma with Cytotoxic T-Lymphocyte Signatures Activated by Chemoradiotherapy

**DOI:** 10.1371/journal.pone.0143804

**Published:** 2015-12-01

**Authors:** Yosuke Tanaka, Kazuhiko Aoyagi, Keiko Minashi, Rie Komatsuzaki, Masayuki Komatsu, Fumiko Chiwaki, Masashi Tamaoki, Takao Nishimura, Naoki Takahashi, Ichiro Oda, Yuji Tachimori, Tokuzo Arao, Kazuto Nishio, Shigehisa Kitano, Kenta Narumi, Kazunori Aoki, Satoshi Fujii, Atsushi Ochiai, Teruhiko Yoshida, Manabu Muto, Yasuhide Yamada, Hiroki Sasaki

**Affiliations:** 1 Gastrointestinal Medical Oncology Division, National Cancer Center Hospital, Tokyo, Japan; 2 Department of Gastrointestinal Oncology and Endoscopy, National Cancer Center Hospital, Tokyo, Japan; 3 Department of Esophageal Surgery, National Cancer Center Hospital, Tokyo, Japan; 4 Department of Experimental Therapeutics, National Cancer Center Hospital, Tokyo, Japan; 5 Department of Translational Oncology, National Cancer Center Research Institute, Tokyo, Japan; 6 Division of Molecular and Cellular Medicine, National Cancer Center Research Institute, Tokyo, Japan; 7 Division of Genetics, National Cancer Center Research Institute, Tokyo, Japan; 8 Fundamental Innovative Oncology Core Center, National Cancer Center Research Institute, Tokyo, Japan; 9 Department of Genome Biology, Kinki University Faculty of Medicine, Osaka, Japan; 10 Department of Clinical Trial Promotion, Chiba Cancer Center, Chiba, Japan; 11 Department of Therapeutic Oncology, Kyoto University, Kyoto, Japan; National Cancer Center Research Institute, JAPAN

## Abstract

Definitive chemoradiotherapy (CRT) is a less invasive therapy for esophageal squamous cell carcinoma (ESCC). Five-year survival rate of locally advanced ESCC patients by definitive CRT were 37%. We previously reported that tumor-specific cytotoxic T-lymphocyte (CTL) activation signatures were preferentially found in long-term survivors. However, it is unknown whether the CTL activation is actually driven by CRT. We compared gene expression profiles among pre- and post-treatment biopsy specimens of 30 ESCC patients and 121 pre-treatment ESCC biopsy specimens. In the complete response (CR) cases, 999 overexpressed genes including at least 234 tumor-specific CTL-activation associated genes such as *IFNG*, *PRF1*, and *GZMB*, were found in post-treatment biopsy specimens. Clustering analysis using expression profiles of these 234 genes allowed us to distinguish the immune-activated cases, designating them as I-type, from other cases. However, despite the better CR rate in the I-type, overall survival was not significantly better in both these 30 cases and another 121 cases. Further comparative study identified a series of epithelial to mesenchymal transition-related genes overexpressed in the early relapse cases. Importantly, the clinical outcome of *CDH2*-negative cases in the I-type was significantly better than that of the *CDH2*-positive cases in the I-type. Furthermore, NK cells, which were activated by neutrophils-producing S100A8/S100A9, and CTLs were suggested to cooperatively enhance the effect of CRT in the *CDH2*-negative I-type. These results suggested that CTL gene activation may provide a prognostic advantage in ESCCs with epithelial characteristics.

## Introduction

Esophageal cancer is an aggressive tumor with a poor prognosis. Common histopathology of esophageal carcinoma varies according to geographical areas. Squamous cell carcinoma, located mostly in the thoracic esophagus, is the major histological type in East Asian countries and a part of Europe, while adenocarcinoma, located in the distal part of the esophagus, is the major histological type in Europe and North America.

Surgery was the traditional standard therapy for localized esophageal squamous cell carcinoma (ESCC); however, outcomes of surgery alone were poor in the early years [1]. While the surgical approach for a better outcome has been investigated [2], favorable results from neoadjuvant chemotherapy or neoadjuvant chemoradiotherapy followed by surgery have been reported from Japan and other countries, with 5-year survival rates of up to 55% [3, 4]. Another treatment option, definitive chemoradiotherapy (CRT), has a curative potential for ESCC even in cases of locally advanced carcinoma [5]. With resectable cases, 5- and 3- year survival rates were 36.8% [6] and 63.8% [7] in two prospective trials, although the treatment outcome is inferior to surgery, and significant late toxicities were occasionally reported [8]. Definitive CRT is regarded as one of the treatment options for localized ESCC.

Gene expression profiles from DNA microarrays have been utilized in the diagnosis and prediction of cancer prognosis. Surgically resected samples have been most often used as subjects for gene expression profiling. Recently, along with an increase in neoadjuvant therapy (in head and neck, esophageal, lung, pancreatic, prostate and breast cancers) and definitive CRT (in esophageal cancer), pre-treatment biopsy samples have become essential for transcriptome analyses as well as genome analyses for identifying prognostic factors and therapeutic targets in the above-mentioned recent modalities.

The activation of an immune response is increasingly recognized as essential for a better prognosis in treating cancer. The presence of tumor infiltrating lymphocytes within the tumor tissue has been shown to be predictive of a prognosis, or effectiveness of treatment in various types of tumors including ESCC [9]. In our previous report on microarray-based gene expression analysis, the preferential activation of immune-related genes was identified in pre-treatment samples from long-term survivors after CRT [10]. The involvement of tumor infiltrating lymphocytes was also shown to be associated with a better prognosis after CRT.

Here we conducted gene expression profiling, using oligonucleotide microarrays of pre- and post-treatment biopsy specimens from 30 ESCC patients treated with definitive CRT and 121 pre-treatment ESCC biopsy specimens. The purpose of the study was to explore the effect of CRT on tumor tissues comparing pre- and post-treatment samples, especially from the perspective of the immune response.

## Materials and Methods

### Clinical samples

All esophageal squamous cell carcinoma (ESCC) specimens were provided by the National Cancer Center Hospital and National Cancer Center Hospital East, after obtaining written informed consent from each patient and approval by the National Cancer Center Institutional Review Board. Patients received definitive chemoradiotherapy (CRT) treatment: protracted infusion of 5-FU 800 to 1000 mg/m^2^/24 hours on days 1 to 4 and 29 to 32, a 2-hour infusion of CDDP 75 to 80 mg/m^2^ on days 1 and 29, and concurrent radiation therapy at a dose of 50.4 to 60 Gy. After the completion of CRT, responders additionally received two courses of chemotherapy every 4 weeks. Treatment response was evaluated 8 weeks after CRT. For the biopsy samples, tumor portions (about 2 mm X 2 mm) of patients before and after (3–4 weeks) treatment were obtained under endoscopy from a margin of the tumor by exclusion of any central necrotic lesions to obtain viable cancer cells. All biopsy samples were immediately exposed to ISOGEN lysis buffer (Nippon Gene Co., Ltd., Toyama, Japan) or frozen with liquid nitrogen, and stored at -80°C until use.

### RNA extraction and microarray analysis

The RNAs of the biopsy samples were isolated by homogenizing them in an ISOGEN lysis buffer followed by phenol extraction and precipitation in isopropanol. The samples were treated with RNase-free DNase I in the presence of RNase inhibitor and re-isolated by the above procedure with ISOGEN lysis buffer. The RNA samples were finally subjected to microarray analysis after quality check by RNA 6000 nano LabChip kit (Agilent Technologies Ltd., CA, USA). Gene expression profiles were obtained from 185 samples: 60 biopsy samples from before and after CRT of 30 identical patients, and 121 before CRT from another ESCC patient set. Total RNAs extracted from the needle biopsy samples were biotin-labeled and hybridized to high-density oligonucleotide microarrays (Human Genome U133PLUS2.0 Array, Affymetrix, Santa Clara, CA, USA) in accordance with the manufacturer’s instructions. The scanned data of the arrays were processed by Affymetrix Microarray Suite version 5.0, which scaled the average intensity of all the genes on each array to a target signal of 1,000 to reliably compare variable multiple arrays. All the microarray data have been deposited in a MIAME compliant database, GEO; the accession number GSE69925.

### Hierarchical clustering and gene selection

Hierarchical clustering and gene selection from microarray data were performed using GeneSpring (Agilent Technologies Ltd., CA, USA), Microsoft EXCEL, and Cluster & TreeView software [11]. Hierarchical clustering is widely used as one of the unsupervised learning methods. For unsupervised clustering, we firstly selected genes with a signal intensity of more than 500 in more than 10% of all samples for clustering, and from these genes, we finally selected more than 2-fold changed genes by comparing the average signal intensity of each gene in more than 10% of the samples. For overexpressed genes in biopsy samples before or after CRT, we first selected genes with a signal intensity of more than 500 in more than 10% of all samples for gene selection, and from these genes, we selected final gene sets by a combination of t-test (*p*<0.05) and 2-fold change between biopsy samples before and after CRT.

### Statistical analysis

The probabilities of overall survival (OS) were estimated using the Kaplan-Meier method, and the log-rank test was used to evaluate the differences between groups. Statistical analyses were performed with EZR version 1.27, which is a graphical user interface for R (The R Foundation for Statistical Computing, version 3.1.1).

## Results

### Patient characteristics and gene expression-based clustering analysis

The locally advanced 30 ESCC patients (stages II-III) at the National Cancer Center Hospital were enrolled in the analysis. Patient median age was 62. Fourteen of the 30 patients were stage II, 16 were stage III. Treatment response was evaluated 8 weeks after CRT. For the post-treatment biopsy samples, tumor portions were obtained 3–4 weeks after treatment under endoscopy from a margin of the tumor by exclusion of any central necrotic lesions to obtain the viable cancer cells. A total of 19 of the 30 patients achieved a complete response (CR) status after CRT alone. Before conducting microarray analyses, we quantified the RNA obtained from pre- and post-treatment biopsy samples. Corresponding to the responsiveness to CRT, a decreasing rate of the RNA amount of the post-treatment sample to the pre-treatment sample in CR cases is relatively higher than that in the non CR (partial response, PR) cases (S1A and S1B Fig). Therefore, we concluded that information about cellular events associated with responsiveness would be revealed by comparative gene expression profiling between pre-and post-treatment samples.

**Fig 1:**
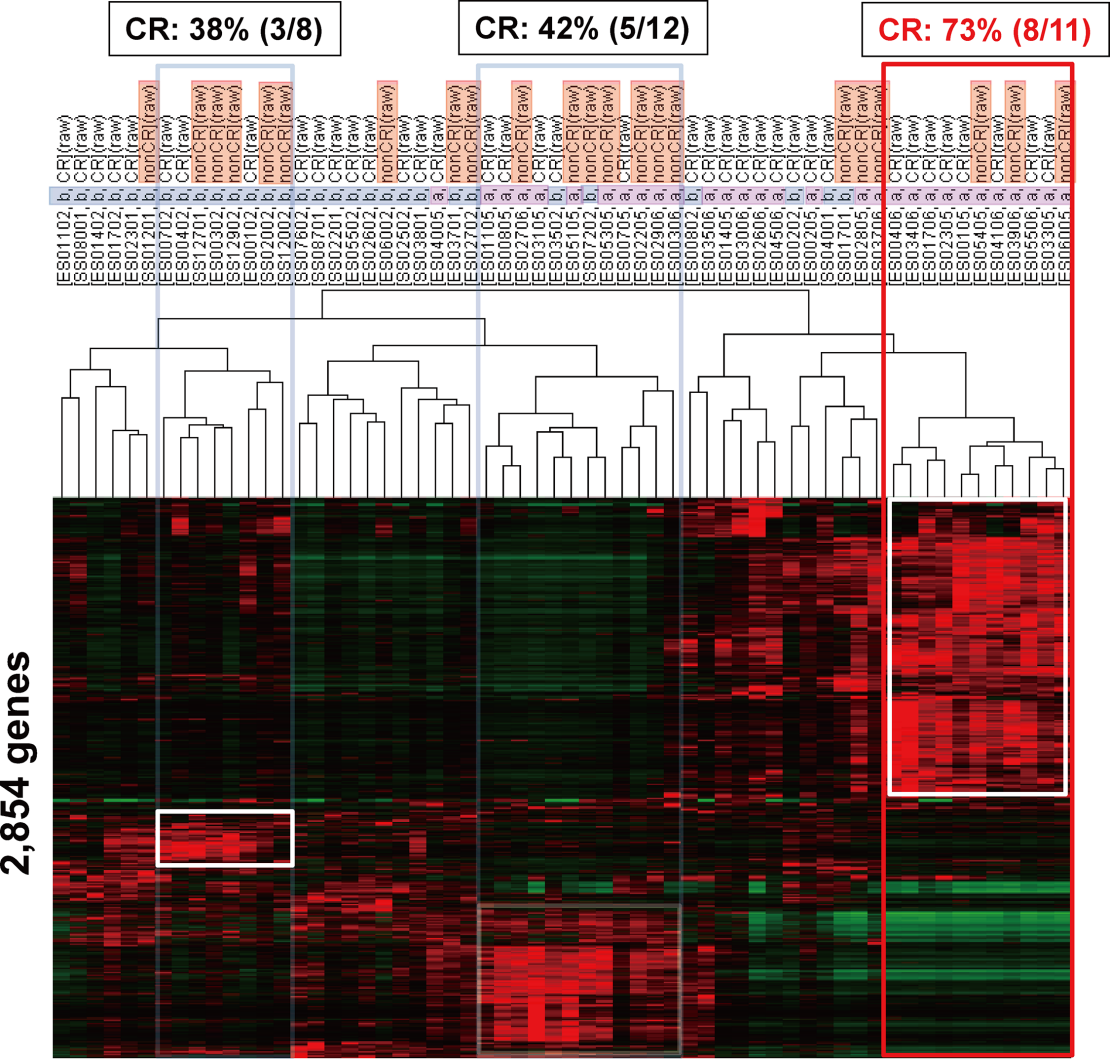
Unsupervised clustering analysis with expression data of the processed 2,854 genes in the 60 ESCC samples treated with definitive CRT. Expression data in all of the 60 samples (A: 30 pre-treatment biopsy samples, B: 30 post-treatment biopsy samples) were analyzed by the Cluster and Treeview programs. Three patient clusters (box) were identified. Among them, one cluster (red) was a good responder with 73% CR.

**Fig 2:**
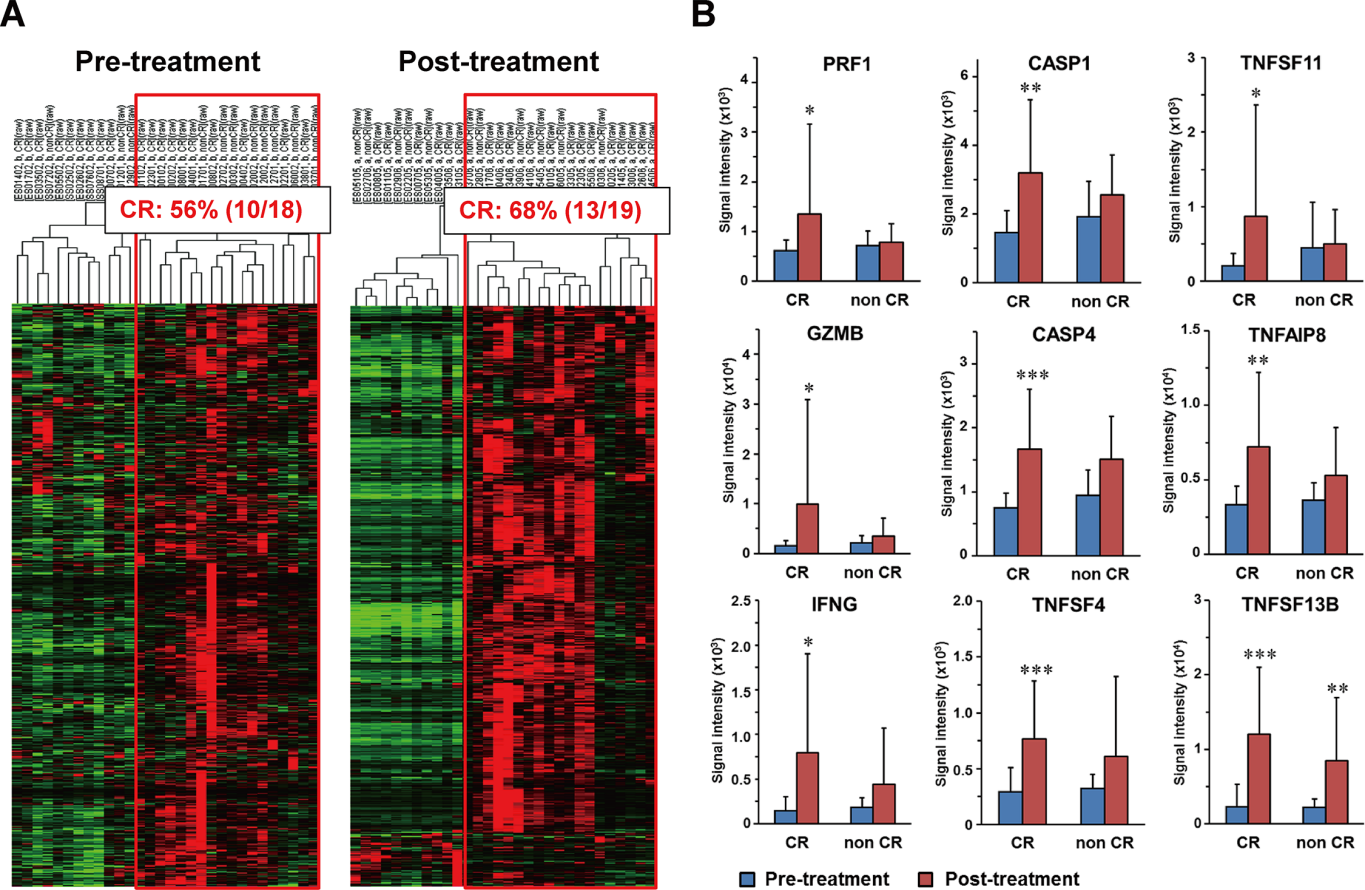
Supervised clustering analysis with expression data of the 999 genes induced by CRT in a good responder cluster. (A) In accordance with the unsupervised clustering analysis with expression data of the processed 2,854 genes, the dendrograms reproducibly showed the presence of a good responder cluster (CR: 56% and 68%) in both pre- and post- treatment samples. (B) Comparison of immune-related gene expression levels between pre- and post- treatment samples. Key genes (*PRF1*, *GZMB*, *IFNG*, *CASPs*, *TNFs*) for the CTL activation, which were included in 234 immune-related genes of the above 999 genes, were upregulated in post-treatment samples especially with CR cases. Pre-treatment samples (blue), post-treatment samples (red). CR: cases with complete response, non CR: cases with non CR (partial response, PR). * *p*<0.05, **p<0.01, ***p<0.001.

We first conducted an unsupervised clustering analysis in 30 pre- and 30 corresponding post-treatment biopsy samples; thus 60 samples using 2,854 processed genes (Materials and Methods). Three intrinsic patient clusters were identified (Fig 1). The CR rate in each patient cluster was 38% (3/8), 42% (5/12), and 73% (8/11), respectively. Thus, the third patient cluster can be regarded as a good responder subtype. We were able to extract 999 and 688 genes, whose expression was significantly high or low after treatment in the 19 CR cases, using Student’s t-test (*p*<0.05) (S1 Table and S2 Table). By the same way, we also extracted 268 overexpressed and 486 underexpressed genes in the 11 non CR (PR) cases (S3 Table and S4 Table). The 999 overexpressed genes in the 19 CR cases included mesenchymal-related genes (*VIM*, *TWIST2*, and *ZEB2*) and cytotoxic T-lymphocyte activation-related genes, while the 688 underexpressed genes included epithelial cell-related genes (*KRTs* and *CDH1*). The 268 overexpressed genes in the 11 non CR (PR) cases included CDK inhibitor and p53-related genes that induce growth arrest in G1 phase, while the 486 underexpressed genes included S phase marker genes (*MCM6*, *CCNA1*, *CCNE2*, *CHEK1*, and *CENPs*). Impressively, among these 4 overexpressed or underexpressed gene sets, most of these 999 genes were identical to the overexpressed genes in the aforementioned good responder (Fig 1, the right patients’ cluster in a red box). Using these 999 genes, clustering analyses of the 30 pre- and 30 post-treatment samples were performed (Fig 2A). Overexpression of these 999 genes was found in 18 pre-treatment and 19 post-treatment samples, respectively. Among these samples, CR cases were included in 56% (10/18) of pre-treatment samples, and in 68% (13/19) of post-treatment samples. Although the 999 genes were selected as highly expressed genes in 19 post-treatment samples from CR cases, overexpression of these genes can be recognized even in pre-treatment samples.

### Clustering with 234 immune-related genes found in the 999 genes

Among the 999 genes, as many as 23% (234 of 999) were revealed to be immune activation-related genes on the basis of Gene Ontology, such as perforin (*PRF1*), granzyme B (*GZMB*), interferon-gamma (*IFNG*), caspases (*CASPs*), and tumor necrosis factors (*TNFs*), suggesting the involvement of activation of cytotoxic T-lymphocytes (CTLs) in a good responder subtype found in this study (S5 Table). Expression levels of 9 representative genes (*PRF1*, *GZMB*, *IFNG*, *CASP1*, *CASP4*, *TNFSF4*, *TNFSE11*, *TNFSF13B*, and *TNFAIP8*) are shown in Fig 2B. These genes were overexpressed significantly in post-treatment samples especially in CR cases. These results suggested that CTL was activated in the CR cases by CRT.

**Fig 3:**
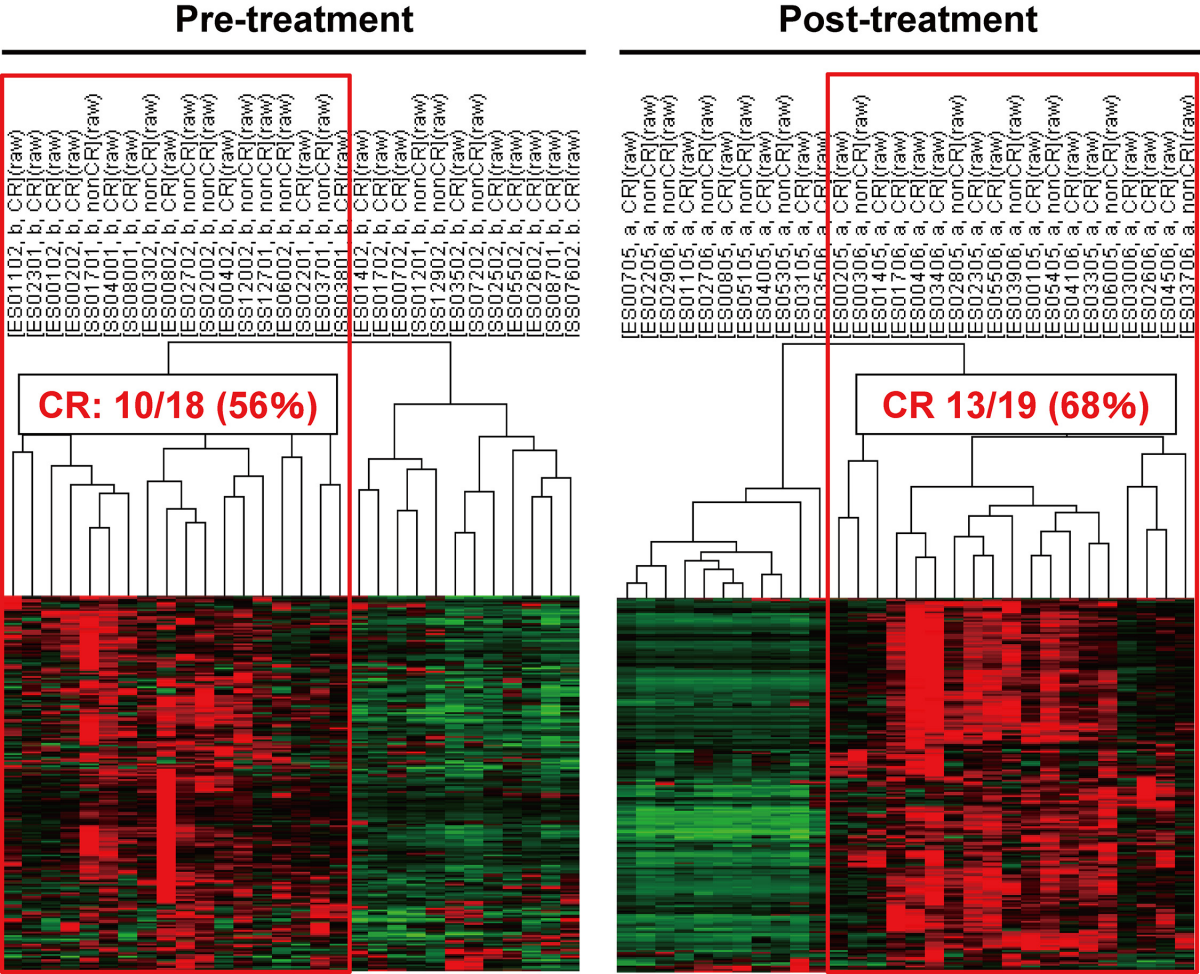
Supervised clustering analysis with expression data of the 234 immune-related genes in the 60 ESCC samples. In accordance with supervised clustering analyses with expression data of the 999 genes (Fig 1A), the dendrograms reproducibly showed the presence of a good responder cluster (CR: 56% and 68%) in each pre- and post- treatment samples. We designated the immune-activated ESCC subtype as I-type.

In order to clarify the effect of immune-related genes on the treatment outcome, we again performed clustering analyses with these 234 immune activation-related genes. The analyses revealed that 18 and 19 cases in pre- and post-treatment samples showed a high expression of the 234 genes (Fig 3). A considerable number of CR cases were included in each patient cluster (56%, 10/18 and 68%, 13/19). The CR rates were exactly the same (68%) when the 999 genes or 234 genes were used in the clustering. Accordingly, the 234 selected genes may still include genes unrelated to immune activation. We designated the immune-activated ESCC subtype as I-type.

### Prognosis of an ESCC subtype with a high expression of the immune activation-related genes in two sets of samples

**Fig 4:**
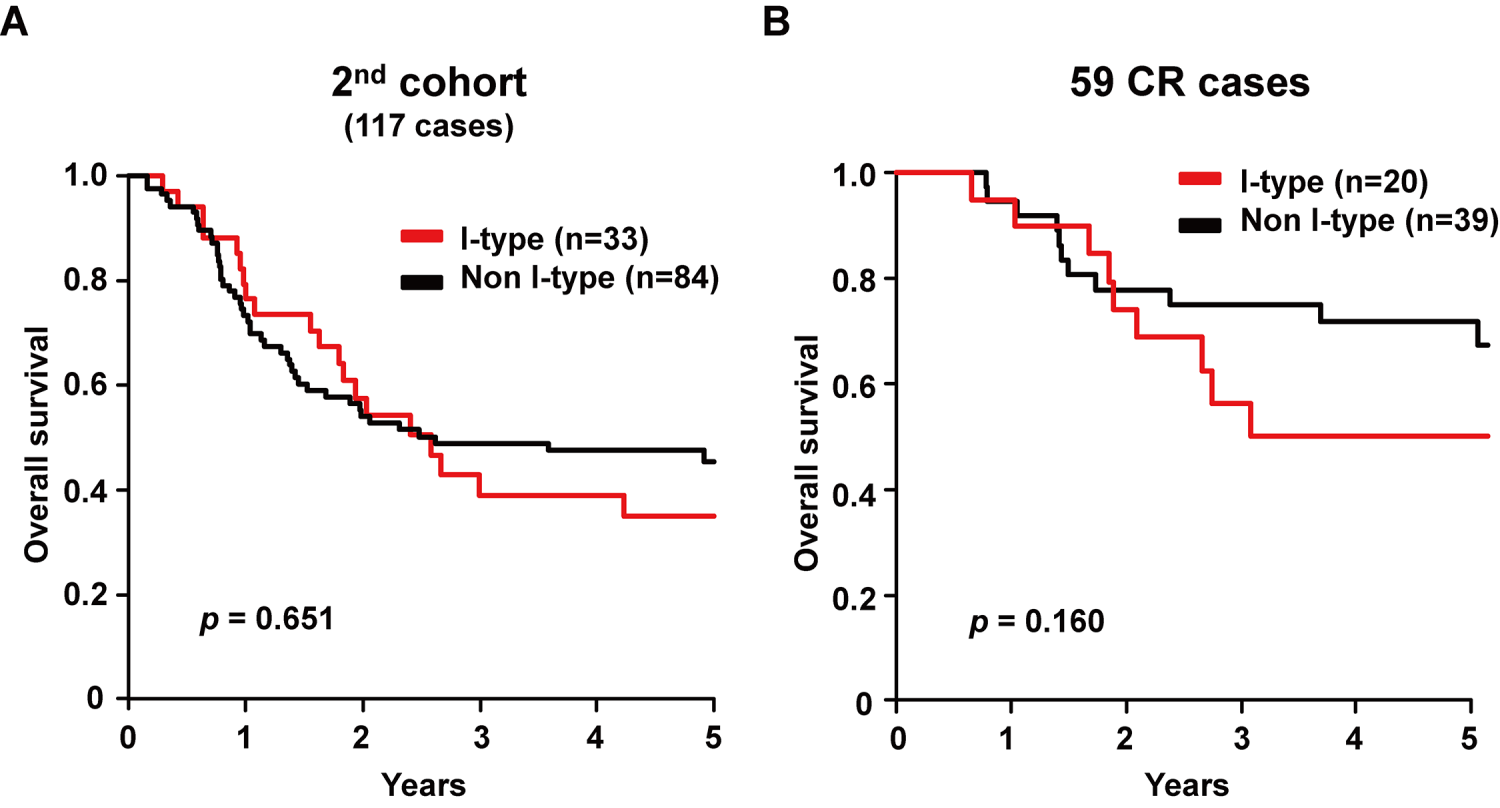
Overall survival in the I-type cases and non I-type cases in 117 cases of the second cohort. The I-type cases did not show better overall survival in both all of the 117 cases (A) and the 59 CR cases (B).

Next, we analyzed the effect of the CRT-induced immune activation on overall survival (OS). Contrary to the better outcome of the CR rate in the I-type, there was no significant difference in OS between 19 I-type cases and 11 non I-type cases, with a 5 year OS of 21% and 58% (*p* = 0.095, S2 Fig). Because we presumed that the case number of 30 might be insufficient to analyze OS, we further investigated another 121 pre-treatment biopsy samples from the National Cancer Center Hospital East in order to increase the statistical power. In the same way, the better result of the CR rate (20/34, 59% vs 39/87, 45%) in 34 I-type cases compared to 87 non I-type cases was observed in these 121 second cohort samples; however, no significant difference was detected in OS between I-type and non I-type (Fig 4A), and even among the 59 CR cases, OS was never better in 20 I-type cases compared with that in 39 non I-type cases (Fig 4B).

### Prognosis of the I-type with epithelial or mesenchymal characteristics

In the second cohort of locally advanced 121 ESCCs, we next compared gene expression profiles between the I-type cases with and without early relapse. A series of epithelial to mesenchymal transition-related genes encoding N-cadherin, collagens, laminins, alpha actin, or fibronectin were identified to be overexpressed in the early relapse I-type cases (S6 Table), whereas squamous epithelial cell marker genes (*MUC1*, *SBSN*, *FLG*, *KRTs*, and *SERPINs*) were overexpressed in the I-type cases without the early relapse (S7 Table). We previously showed that the intestinal-type with epithelial characteristics and the diffuse-type with mesenchymal characteristics can be clearly distinguished using the ratio of *CDH1* (E-cadherin) mRNA and *CDH2* (N-cadherin) mRNA in gastric cancer [12]. *CDH1* is known to be a typical epithelial cell marker, while *CDH2* is a mesenchymal cell marker. In ESCCs, the *CDH1* mRNA level is very low. Accordingly, we used the *CDH2* mRNA as a single marker to distinguish the mesenchymal phenotype from the epithelial phenotype in this study. Affymetrix microarray provides us with three kinds of detection calls for each gene probe including P (presence), M (marginal), and A (absence). Out of 121 cases, 117 cases were used in the next study because we eliminated 4 cases with the M call in the *CDH2* mRNA signature. We summarized tumor stages, CR rate, and 1 year relapse free (RF) rate of the 117 cases (S8 Table). In 33 I-type cases, the CR rate and the RF rate of 15 *CDH2*-negative cases with the A call was 80% and 64%, while that of 18 *CDH2*-positive cases with the P call was 44% and 11%, respectively. In 84 non I-type cases, the CR rate and the RF rate of 67 *CDH2*-negative cases was 52% and 42%, while that of 17 *CDH2*-positive cases was 18% and 12%, respectively. The number of stage II cases was approximately half the number of stage III cases in all the 117 cases, in 82 *CDH2*-negative cases, in 35 *CDH2*-positive cases, in 84 non I-type cases, in 67 *CDH2*-negative non I-type cases, and in 17 *CDH2*-positive non I-type cases. Among 33 I-type cases, 15 *CDH2*-negative I-type cases, and 18 *CDH2*-positive I-type cases, the number of stage II (60%) was higher than that of stage III (40%) only in the 15 *CDH2*-negative I-type cases.

**Fig 5:**
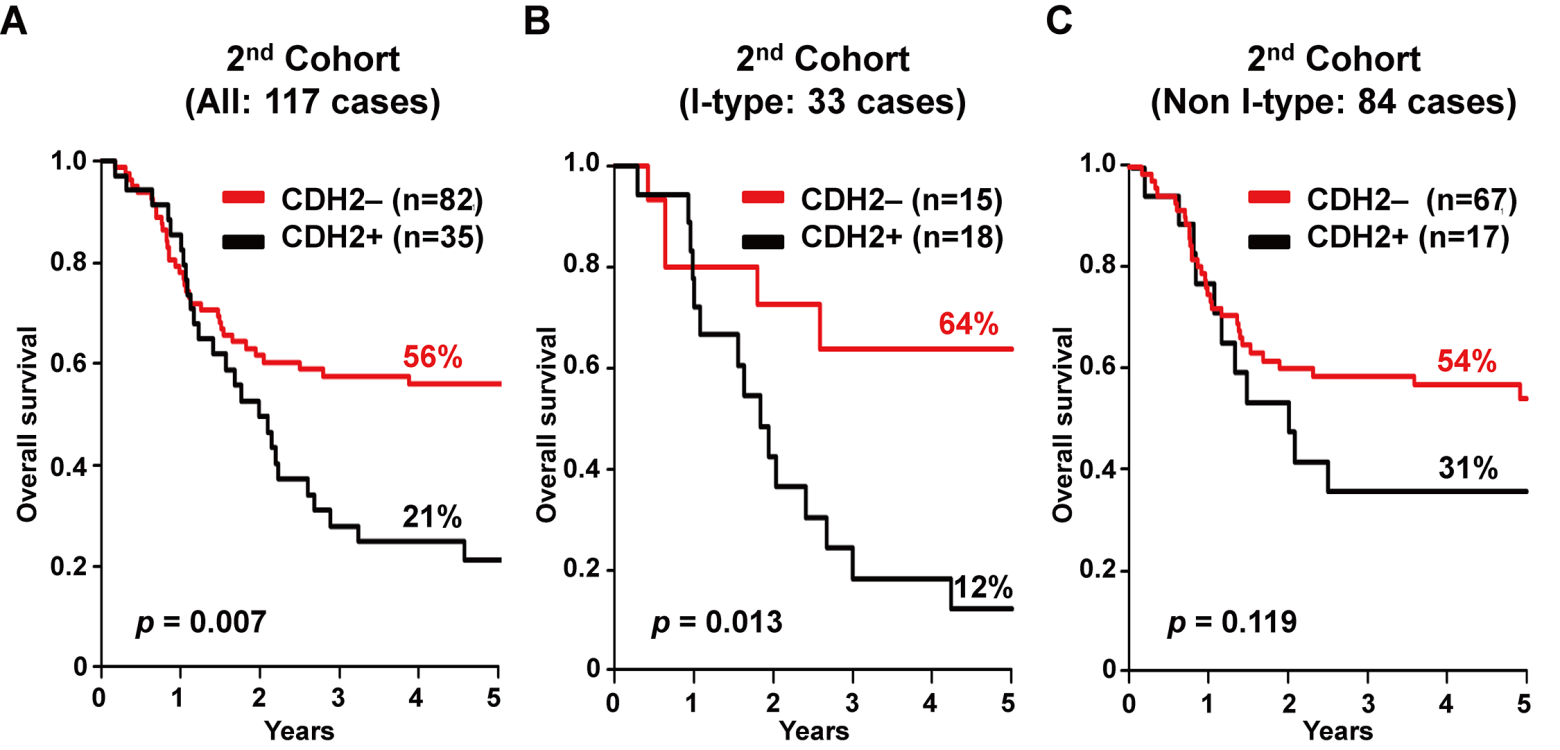
Overall survival in the *CDH2*-negative epithelial-type cases and *CDH2*-positive mesenchymal-type cases in 117 cases of the second cohort. (A) In all of the 117 cases, 82 *CDH2*-negative cases showed better overall survival (OS) compared with 35 *CDH2*-positive cases (56% vs 21% in 5 year OS rate). (B) Especially, in 33 I-type cases, 15 *CDH2*-negative cases showed much better OS compared with 18 *CDH2*-positive cases (64% vs 12% in 5 year OS rate). (C) In 84 non I-type cases, 67 *CDH2*-negative cases showed better OS compared with 17 *CDH2*-positive cases (54% vs 31% in 5 year OS rate).

Coincident with these results, in all of the 117 cases from the second cohort, OS in the 82 *CDH2*-negative epithelial-type (E-type) cases was significantly better than the 35 *CDH2*-positive mesenchymal-type (M-type) cases, with a 5 year OS rate of 56% vs 21% (*p* = 0.007, Fig 5A). Among the 33 I-type cases, the 15 *CDH2*-negative E-type cases had significantly better OS compared with the 18 *CDH2*-positive M-type cases, with a 5 year OS rate of 64% vs 12% (*p* = 0.013, Fig 5B). Among the 84 non I-type cases, the 67 *CDH2*-negative E-type cases had not significantly but clearly better OS compared with the 17 *CDH2*-positive M-type cases, with a 5 year OS rate of 54% vs 31% (*p* = 0.119, Fig 5C).

In summary, in *CDH2*-negative E-type cases, the CR, RF, or OS rate of I-type (80, 64, or 64%, respectively) was 28, 22, or 10 points higher than that of non I-type (52, 42, or 54%, respectively). However, in *CDH2*-positive M-type cases, the RF or OS rate of I-type (11, 31%) was same or lower than that of non I-type (12, 12%). although the CR rate of I-type (44%) was 26 points higher than that of non I-type.

Recently, we reported that proinflammatory proteins S100A8/A9 via interaction with RAGE enhanced the activation of NK cells, which produce interferon-gamma (IFN-γ), and significantly suppressed tumor growth. Furthermore, the numbers of CD4^+^ and CD8^+^ T cells infiltrated in S100A8/A9-exprssing tumors were significantly increased in NK cell-depleted mice (Narumi et al., 2015). Interestingly, in epithelial I-type ESCCs without relapse compared with mesenchymal I-type with early relapse, *S100A8* and *S100A9* were found to be overexpressed 3.3- and 5.2-fold, respectively (S7 Table). These data suggested that NK cells and CTLs cooperatively enhance the effect of CRT in the epithelial I-type ESCCs.

## Discussion

The results obtained from our clustering analysis on gene expression profiles clearly indicated that increment of mRNA levels of CTL activation-related genes is induced by CRT and is related to a better antitumor response by CRT in I-type ESCCs which show overexpression of these genes before CRT (Figs 2 and 3). Most of these genes are considered to be involved in the activation of CTLs. In the I-type ESCCs, the CTL activation may enhance the elimination of tumor cells for at least several months, because treatment response was evaluated 8 weeks after CRT. The prognostic value of immunoreaction during cancer treatment has been investigated by evaluating tumor infiltrating immune-related cells in various types of cancers [13, 14]. Tumor infiltrating immune cells such as cytotoxic T-lymphocytes (CTLs), helper T cells, macrophages, and regulatory T cells are regarded as the surrogate marker candidates of immune reaction. Accordingly, the relationships between the infiltration status of these immune-related cells and the prognosis or therapeutic response have been assessed; however, the results of various cell types except CTLs are as yet not consistent, especially for the prognostic value of regulatory T cells [15]. In ESCC, CTLs (CD8^+^ T cells) infiltration into a tumor has been reported to be a good prognostic factor by two groups [16, 17]. Increased infiltration of CTLs after chemotherapy has also been suggested in ESCC [18]. The immune activating effect of radiation via enhancement of activity of CTLs has also been reported [19, 20]. The mechanism of immune activation after chemotherapy is attributed to the release of tumor associated antigens through the destruction of tumor cells, or the suppressive effect of chemotherapy on the immune inhibitory cells, such as regulatory T cells or myeloid-derived suppressor cells (MDSCs) [21].

However, in this study, no direct benefit of immune activation for OS could be observed even in the cases with better therapeutic response (S2 Fig and Fig 4). In the process of scrutinizing data for the cause of the poor prognosis by gene expression profiling, we found that the cases with early relapse had mesenchymal characteristics (data not shown). Mesenchymal characteristics are known to be related with poor clinical outcomes, which are often explained by EMT [22]. With the use of a *CDH2* expression status as a single marker to distinguish the orientation for the mesenchymal or epithelial phenotypes, we identified an innate poor response and prognosis in the mesenchymal type. The *CDH2*-negative cases in the I-type had significantly better OS compared with the *CDH2*-positive cases, while *CDH2*-positive cases showed poor OS irrespective of the status of immune activation (Fig 5). More importantly, in *CDH2*-negative cases, the CR, RF, or OS rate of I-type (80, 64, or 64%, respectively) was 28, 22, or 10 points higher than that of non I-type (52, 42, or 54%, respectively); however: in *CDH2*-positive cases, the RF or OS rate of I-type (11, 31%) was same or lower than that of non I-type (12, 12%), although the CR rate of I-type (44%) was 26 points higher than that of non I-type (S8 Table and Fig 5). Therefore, these results suggested that the CTL-activation was beneficial for the epithelial type, but alleviated the effect in the mesenchymal type.

Recently, remarkable advances have been recognized in the cancer immunotherapy field, such as clinical success in anti-CTLA-4 antibody or anti-PD1 antibody [23], and the upcoming T cell therapy including chimeric antigen receptor-T cells [24]. Preclinical models already showed the synergistic effect of the combination of radiation and these immunomodulating agents [25]. Our results may imply the possibility that the addition of an immunotherapy that can augment the function of CTL will further improve the outcomes of CRT for ESCC, and that effects will be more beneficial for tumors with epithelial characteristics than for those with mesenchymal ones. Overexpression of *S100A8* and *S100A9* in epithelial I-type ESCCs without relapse may activate a cellular immunity including NK cells and CTLs, which produce IFN-γ (S7 Table). Thus, NK cells and CTLs are thought to enhance cooperatively the effect of CRT in the epithelial I-type ESCCs. The *in vivo* source of S100A8/A9 in tumor tissue is tumor cells and myeloid cells such as neutrophils [26]. Recently it is reported that neutrophils crosstalk with NK cells: the NK cell-derived IFN-γ modulates the survival and functional responses of neutrophils, and conversely, neutrophil-derived IL-15 and IL-18 are essential for NK cell activation and proliferation [27]. Interestingly, we found that a transcriptional repressor SIM2, which induces differentiation of ESCC cells [28] and overexpresses in the epithelial I-type ESCCs (S7 Table), downregulated strongly both *S100A8* and *S100A9* in a three-dimensional culture of ESCC cells (data not shown). Therefore, S100A8/A9 may be provided by myeloid cells including neutrophils but not by tumor cells in ESCC tissues. Further clarification of the reason why the mesenchymal I-type shows poor prognosis by CRT is something that will have to remain for future study. Although immunohistochemistry (IHC) is a good way to show the presence of the I-type and the evidences for CTL activation or NK activation, biopsy samples are often very small (2 mm X 2 mm) to show the significant difference. Therefore, extensive IHC using multiple sections is required in the future. As the number of cases in our present study is limited, a large cohort study is also needed.

Lastly, we hold that this paper is the first report for the identification of a prognostic factor *CDH2* and for showing the presence of a subtype with immune activation signature through the use of expression profiles of biopsy samples from ESCC patients who received definitive CRT.

## Supporting Information

S1 Fig Comparison of RNA amount between pre- and post-treatment biopsy samples in 19 CR and 11 non CR cases. (A) After elimination of a maximum and minimum value from each case, the bar graphs in 17 CR and 9 non CR cases were shown. (B) The boxplots were shown. The median of 17 CR cases is 42.0μg, while that of 9 non CR cases is 65.2μg.
(TIF)

S2 Fig Overall survival in the immune-activated type (I-type) cases and non I-type cases in 30 cases of the first cohort. Although distinct traits of the CTL activation by CRT were found in the I-type cases, unexpectedly, this type did not show better overall survival.
(TIF)

S1 Table 999 overexpressed genes in 19 post-treatment CR cases. (XLSX)

S2 Table 688 underexpressed genes in 19 post-treatment CR cases. (XLSX)

S3 Table 268 overexpressed genes in 11 post-treatment non CR cases. (XLSX)

S4 Table 486 underexpressed genes in 11 post-treatment non CR cases. (XLSX)

S5 Table 234 immune-activation related genes overexpressed in 19 post-treatment CR cases. (XLSX)

S6 Table 275 overexpressed genes in I-type ESCCs with recurrence within 1 year. (XLSX)

S7 Table 193 overexpressed genes in I-type ESCCs with no recurrence for 1 year. (XLSX)

S8 Table Stage, CR rate and 1 year relapse free rate of 117 ESCC patients received definitive CRT. (XLSX)
