# Supplementary figures and images for: Discovery of a Good Responder Subtype of Esophageal Squamous Cell Carcinoma with Cytotoxic T-Lymphocyte Signatures Activated by Chemoradiotherapy
Source: PLoS One. 2015 Dec 1;10(12):e0143804. doi: 10.1371/journal.pone.0143804 (PMC4666638; doi:10.1371/journal.pone.0143804)

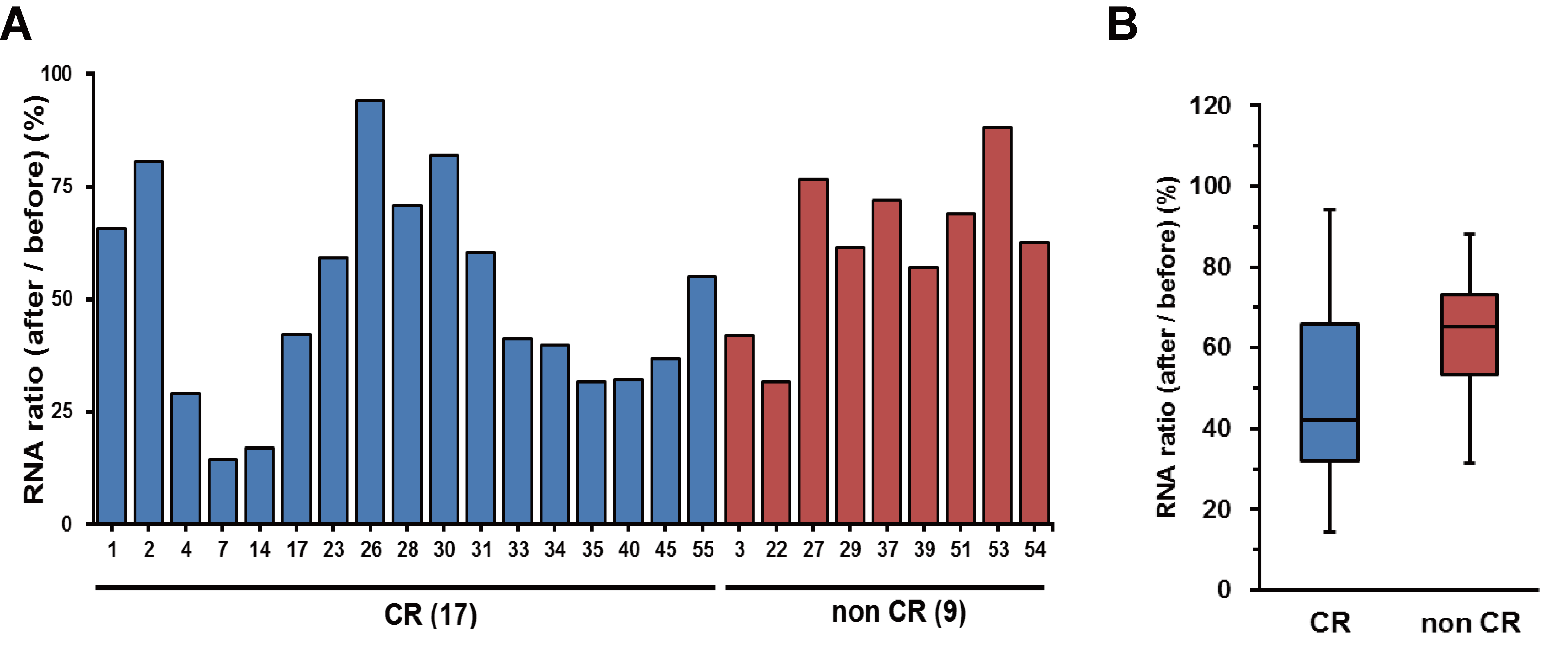

Supplement: S1 Fig — (A) After elimination of a maximum and minimum value from each case, the bar graphs in 17 CR and 9 non CR cases were shown. (B) The boxplots were shown. The median of 17 CR cases is 42.0μg, while that of 9 non CR cases is 65.2μg. (TIF) [file pone.0143804.s001.tif]

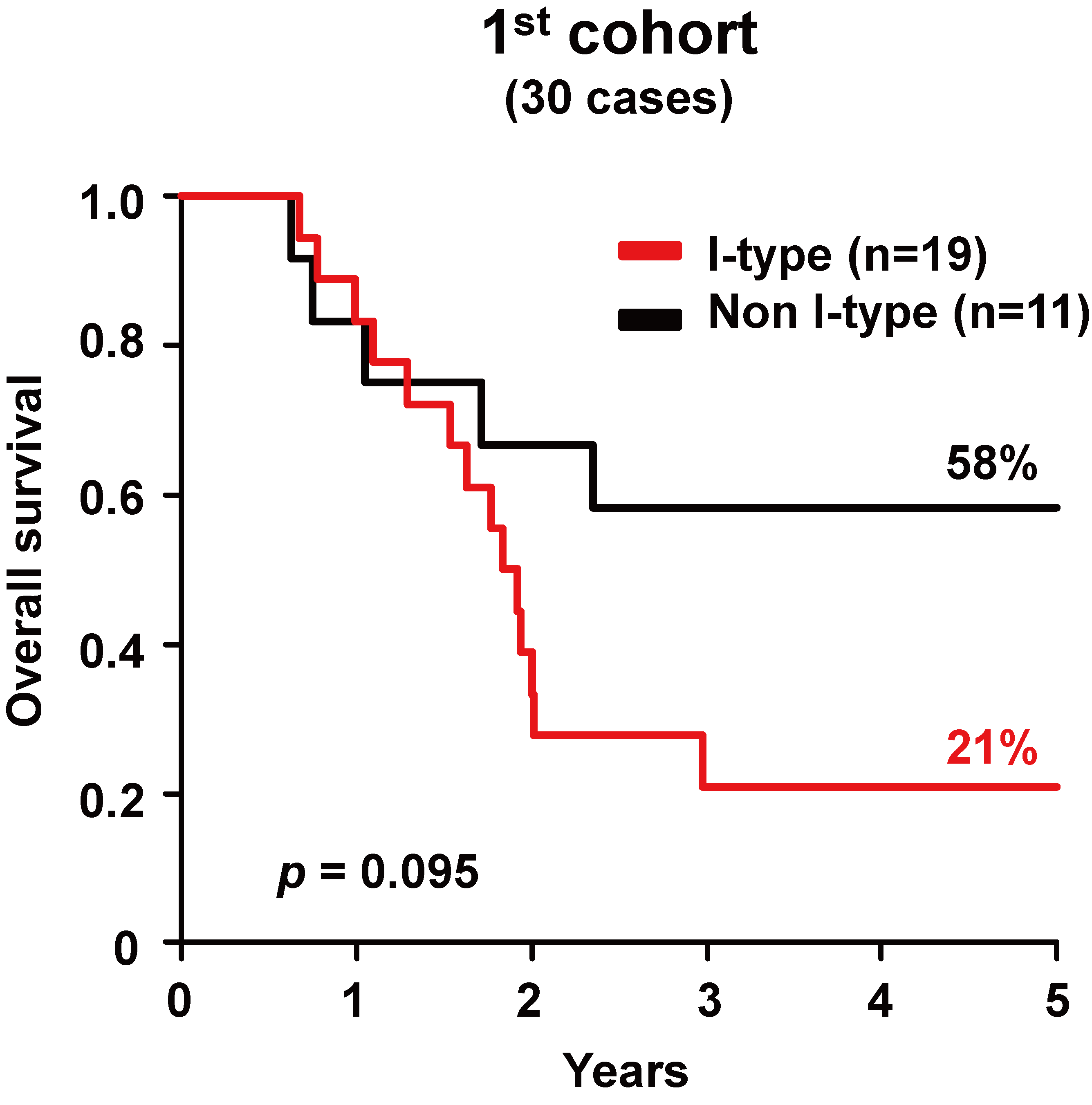

Supplement: S2 Fig — Although distinct traits of the CTL activation by CRT were found in the I-type cases, unexpectedly, this type did not show better overall survival. (TIF) [file pone.0143804.s002.tif]
